# Therapeutic challenge in traumatic arteriovenous fistula of the left lower limb requiring open repair

**DOI:** 10.1590/1677-5449.202301772

**Published:** 2025-07-30

**Authors:** Carlos Eduardo Barbosa Zan, Fábio Husemann Menezes

**Affiliations:** 1 Universidade Estadual de Campinas – UNICAMP, Campinas, SP, Brasil.

**Keywords:** arteriovenous fistula, open treatment, cardiac insufficiency

## Abstract

We report the case of a 38-year-old man with a traumatic arteriovenous fistula between the left common femoral artery and vein, resulting from a gunshot wound sustained 20 years earlier. He began presenting symptoms, including a venous stasis ulcer and cardiac dysfunction, 4 years ago. Computed tomography revealed the presence of a fistula and downstream dilation of the iliac vessels and inferior vena cava, the latter measuring up to 37.5 mm. Given the patient’s complex vascular anatomy and overall good clinical condition, an open surgical approach was selected, resulting in excellent outcomes. Due to the risk of deep vein thrombosis and pulmonary thromboembolism, placement of an inferior vena cava filter and initiation of long-term anticoagulation were also performed. Surgery proved to be the definitive treatment, resolving both heart failure and ulcer, and restoring the patient’s quality of life. The open approach was an excellent treatment option, considering the patient’s young age, anatomical distortion, and the unavailability of adequate endovascular materials.

## INTRODUCTION

An arteriovenous fistula (AVF) is an irregular and permanent communication between an artery and a vein.^1^ Penetrating trauma is the most common cause of traumatic AVF, followed by iatrogenic injuries and blunt trauma. Pathophysiological and structural changes may occur, with blood flowing directly from an artery to a vein, resulting in characteristic physical findings and clinical repercussions for the patient. On physical examination, patients may exhibit a palpable thrill and an audible bruit, caused by turbulent blood flow, while peripheral pulses may be diminished or absent. Other signs in the affected limb include pallor, cyanosis, edema, dermatitis, and, in more advanced and severe cases, intermittent claudication, venous stasis ulcers, and gangrene. Systemic changes, such as cardiac rhythm disturbances or functional impairment, may also occur.^2^

Only about 2% of traumatic AVFs resolve spontaneously,^3^ meaning that surgical correction is required in virtually all cases. Endovascular treatment is the preferred approach; however, in cases with unfavorable anatomy, open surgical repair should be considered.^4^

We report the case of a patient with a traumatic AVF of the left lower limb for 20 years, who had been symptomatic for the past 4 years and was treated via open surgery. The study was approved by the National Research Ethics Committee (approval: 3200843; CAAE: 74856423.3.0000.5404).

## CASE REPORT

### PART I: CASE PRESENTATION

A 38-year-old man presented with complaints of pain, edema, dermatitis, and a venous stasis ulcer on the lateral aspect of the left leg for the past 4 years. Due to these symptoms, he was unable to perform daily activities or work. The patient had been previously healthy and worked in construction. He had sustained a gunshot wound 20 years earlier, with an entry point in the left inguinal region and an exit wound in the ipsilateral buttock. At the time, he was treated at another hospital, where the bullet was removed, and he was discharged without undergoing any imaging studies or outpatient follow-up.

**Figure 1:**
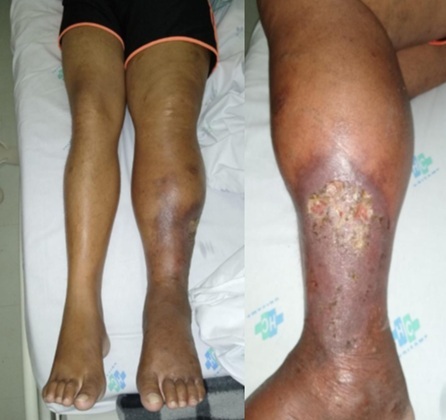
Digital image showing edema, hemosiderin-stained dermatitis, and a venous stasis ulcer on the lateral aspect of the patient’s left leg.

On physical examination, he presented with a pulsatile mass in the left inguinal region, associated with a palpable thrill and audible bruit; edema involving the entire limb, from the upper thigh down, which significantly impaired ambulation; varicose veins and a stasis ulcer on the lateral aspect of the left leg; and presence of only the femoral pulse, with no signs of ischemia (Figure 1). The patient denied symptoms suggestive of heart failure, such as orthopnea or paroxysmal nocturnal dyspnea. However, he did present with painful hepatomegaly, which is suggestive of heart failure.

**Figure 2:**
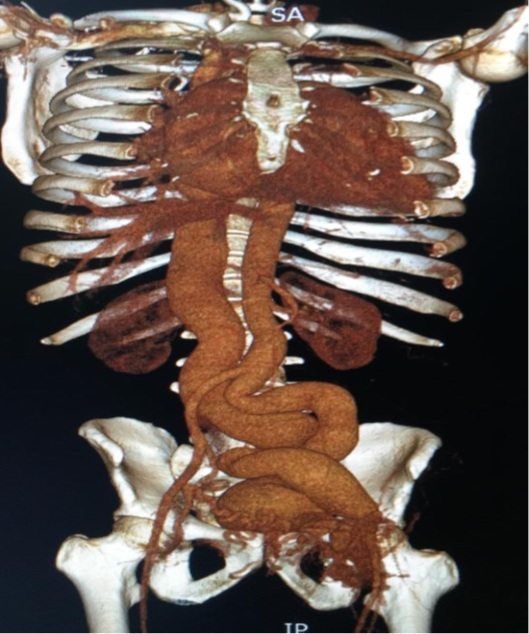
Three-dimensional reconstruction from computed tomography angiography showing dilation of the iliac vessels, inferior vena cava, intrapelvic veins, and veins of the left inguinal region.

**Figure 3:**
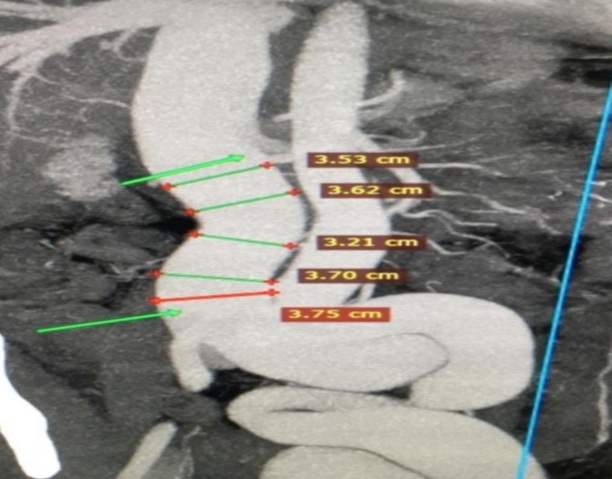
Computed tomography angiography showing dilation of the inferior vena cava measuring up to 37.5 mm in diameter at the iliac vein confluence.

A computed tomography (CT) angiography was performed, which revealed an AVF between the left common femoral artery (LCFA) and the left common femoral vein (LCFV), with downstream dilatation of the iliac vessels and the inferior vena cava (IVC), the latter measuring approximately 37.5 mm at the junction of the iliac veins (Figures 2 and 3). During hospitalization, atrial flutter was identified on electrocardiogram without hemodynamic instability, and high-output heart failure was confirmed on echocardiography. There was decompensation of heart failure, requiring medical management and electrical cardioversion to restore sinus rhythm.

### PART II: WHAT WAS DONE?

Subsequently, treatment of the AVF was performed. Open surgical repair was chosen due to the size of the vessels, the unavailability of adequate endovascular materials, and the fact that the patient was young, otherwise healthy, and in good clinical condition. The left inguinal region was accessed via a transverse inguinotomy, with isolation of the LCFA and partial isolation of the LCFV, given the difficulty of dissection and the presence of adhesions. Due to the caliber of the vessels, the initial plan was to insert and inflate an occlusion balloon in the CFA, above the AVF, in order to reduce blood flow to the vein and allow for AVF repair. However, the inflated balloon was unable to fully occlude the artery. During dissection, the LCFV was injured in an area difficult to clamp with surgical instruments, and bleeding was controlled by digital compression using the index finger and thumb.

**Figure 4:**
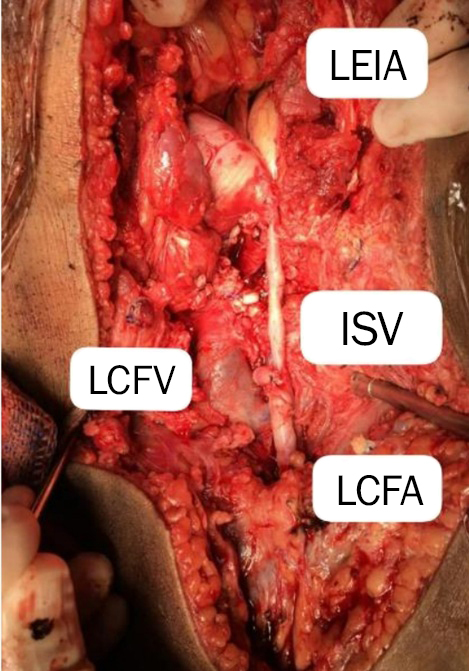
Intraoperative view – Digital image showing the end-to-side anastomosis between the LEIA and LCFA using a reversed ipsilateral great saphenous vein. Note the dilation of the LCFM. LCFA = left common femoral artery; LEIA = left external iliac artery; LCFV = left common femoral vein; ISV = inverted saphenous vein.

It was then decided to open the inguinal canal and the left abdomen, gaining retroperitoneal access, and to surgically isolate and clamp the left external iliac artery (LEIA) and the left external iliac vein, thereby achieving bleeding control. Next, the AVF was repaired with a continuous 5-0 polypropylene suture, resulting in immediate cessation of the thrill. Due to the prolonged course of the fistula and associated fibrosis, the walls of the artery and vein were adhered, making dissection impossible. Therefore, the AVF had to be repaired including both vessel walls. The anterior wall of the vein, which was injured during dissection, was also sutured with a continuous 5-0 polypropylene suture. End-to-side anastomosis was performed between the LEIA and the LCFA, using a reversed ipsilateral great saphenous vein as the conduit of choice, due to arterial wall compromise from the prolonged AVF and the need for joint suturing of the arterial and venous walls to repair the fistula (Figure 4).

An estimated 4,000 mL of blood was lost during the procedure, with the most critical event being the injury to the LCFV. Nonetheless, the patient remained hemodynamically stable, requiring intraoperative transfusion of blood products, administration of crystalloid solutions, and low-dose norepinephrine.

Placement of an IVC filter had been scheduled for the same surgical procedure; however, this was not feasible due to the diameter of the IVC. Full anticoagulation was initiated with subcutaneous enoxaparin at a dose of 1 mg/kg every 12 hours, with a follow-up CT scheduled in the weeks following surgery to reassess the diameter of the IVC.

**Figure 5:**
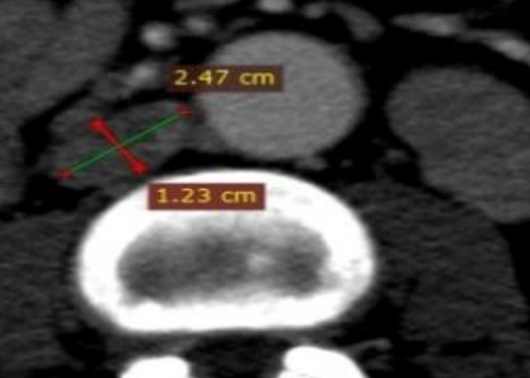
Transverse slice from computed tomography angiography showing the inferior vena cava measuring 24.7 mm in its largest diameter, 3 weeks after surgery.

The patient was extubated on the first postoperative day, the vasoactive medication was discontinued on the second day, and the patient was discharged from the ICU on postoperative day four. In the ward, full anticoagulation was maintained, along with the use of compression stockings, limb elevation while lying down, and dressing of the ulcer. Additionally, early ambulation was encouraged. There was significant improvement in symptoms, mobility, and ulcer healing. CT was repeated in the third postoperative week, revealing a significant reduction in the dilatation of the iliac veins and IVC, with the IVC now measuring 24.7 mm at its widest diameter (Figure 5). This allowed for the successful implantation of a nonremovable Denali™ infrarenal IVC filter (33 mm) via the right common femoral vein, under local anesthesia and without complications. The patient was discharged on postoperative day 23 with a prescription for oral rivaroxaban 20 mg/day, use of compression stockings, and referral for outpatient follow-up.

**Figure 6:**
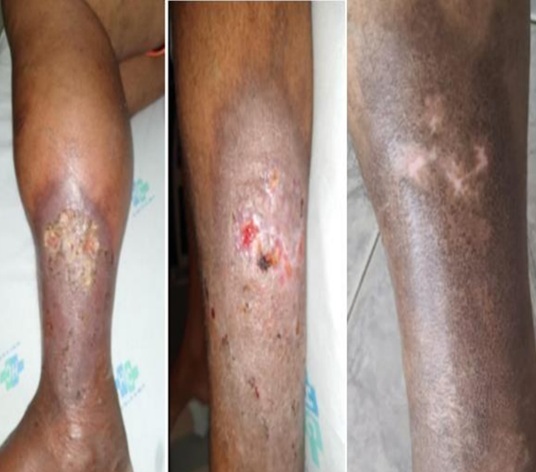
Digital image showing the progression of the venous stasis ulcer on the lateral aspect of the patient’s left leg. From left to right: before surgery, 3 weeks postoperatively, and 3 months postoperatively.

At follow-up visits during the first and third postoperative months, the patient was pain-free, walking normally, without painful hepatomegaly, and in stable sinus rhythm. The ulcer had completely healed by the third month (Figure 6). By the sixth month, the patient remained asymptomatic, had returned to work, and his echocardiogram was normal. Ultrasound revealed graft occlusion and venous dilatation, but with good distal perfusion. Plain radiography confirmed the IVC filter was well positioned. Anticoagulation with rivaroxaban and continuous outpatient monitoring were maintained.

## DISCUSSION

Penetrating injuries are reported as the leading cause of traumatic AVFs. According to Robbs et al., in a study involving 202 patients with traumatic AVFs, penetrating trauma represented 98% of cases, with gunshot wounds accounting for 26% of them. Among those patients, only three developed heart failure, and all had pre-existing cardiomegaly.^5^ This finding highlights the rarity of cardiac involvement. The patient in this report was previously healthy and had no prior cardiac abnormalities. It is likely that the prolonged duration of the AVF led to hemodynamic changes that resulted in cardiac repercussions, requiring pharmacologic management and electrical cardioversion. Surgical intervention is the definitive treatment in these cases. Surgery has been shown to resolve cardiac repercussions, with ligation being the only definitive treatment capable of improving patient survival.^3^

The increased blood flow redirected to the vein leads to elevated intravascular pressure, venous dilatation, valvular insufficiency, blood stasis, hyperpigmentation of the skin, and, in advanced cases, ulceration. After surgery, ulcer healing was observed, along with improved edema, pain, mobility, and overall quality of life. In line with previous studies, healing of venous insufficiency ulcers caused by AVF is easily achieved after surgery.^2^

Endovascular treatment is generally the best option for vascular injuries, especially in complex and potentially high-risk procedures. Open repair, on the other hand, carries significant morbidity and mortality, since elevated venous pressure and inflammation of the surrounding tissues create a hostile local environment. Large collateral vessels with complex venous anatomy are often involved, which can lead to massive hemorrhage^4^ and even death.

In this case, the patient’s anatomy was very unfavorable for endovascular treatment, with extremely dilated and tortuous vessels. Several prosthesis suppliers were contacted, but none had materials large enough for the patient’s vessels. For this reason, and because the patient was young and otherwise healthy, an open surgical approach was chosen. The procedure was challenging due to extreme local adhesions, especially involving the LCFV, as well as marked dilation and tortuosity of the vessels. The LCFV injury was the main cause of intraoperative bleeding, requiring volume resuscitation with crystalloids, transfusion of blood products, and use of norepinephrine. Considering the patient’s clinical condition, the intraoperative response to these measures was favorable, with excellent postoperative recovery.

Due to the risk of deep vein thrombosis and pulmonary embolism – events that occur primarily within the first 30 postoperative days^6^ – full anticoagulation with subcutaneous enoxaparin was initiated, and IVC filter placement was performed after reduction of the IVC diameter, still during hospitalization. At discharge, rivaroxaban 20 mg/day was prescribed. This medication is an oral anticoagulant that acts by directly inhibiting factor Xa. Studies such as the Einstein Extension and Einstein Choice have shown that its prolonged use for up to 12 months carries a low risk of major bleeding.^7,8^ As the patient had a low bleeding risk, excessive venous dilatation, and a nonremovable IVC filter, the initial decision was to use rivaroxaban at the therapeutic dose. During follow-up, the possibility of reducing the diameter of the veins and lowering the rivaroxaban dose to 10 mg/day (prophylactic dose) will be evaluated between 6 and 12 months after the procedure.

According to the Brazilian Guidelines on the Diagnosis and Management of Traumatic Vascular Injuries, vascular trauma of the limbs in hemodynamically stable patients with an ankle-brachial index (ABI) below 0.9 requires imaging, with CT angiography being the gold standard. In cases of penetrating trauma with an ABI ≥ 0.9 and no signs of vascular injury, discharge without imaging is possible, provided outpatient follow-up is ensured due to the potential for delayed manifestations.^9^

One of the key lessons from this case is that the surgeon must not overlook the diagnosis of AVF in cases of penetrating trauma along the course of the vessels – even in the presence of palpable pulses and absence of external bleeding – to avoid late complications. Performing CT angiography and ensuring outpatient follow-up are essential for early and late diagnosis, respectively.

It is concluded that ligation of the traumatic AVF was the definitive treatment for clinical repercussions such as ulceration and heart failure, restoring the patient’s quality of life. Open surgery proved to be an excellent treatment option, given the patient’s young age, distorted vascular anatomy, and the unavailability of adequate endovascular materials.
