# Supplementary material for: Therapeutic challenge in traumatic arteriovenous fistula of the left lower limb requiring open repair
Source: J Vasc Bras. 2025 Jul 30;24:e20230177. doi: 10.1590/1677-5449.202301772 (PMC12419745; doi:10.1590/1677-5449.202301772)
Supplement: Translation

# Translation: Desafio terapêutico em fístula arteriovenosa traumática de membro inferior esquerdo com necessidade de correção aberta

**DOI:** 10.1590/1677-5449.202301771

**Authors:** Carlos Eduardo Barbosa Zan, Fábio Husemann Menezes

**Affiliations:** 1 Universidade Estadual de Campinas – UNICAMP, Campinas, SP, Brasil.

**Keywords:** fístula arteriovenosa, tratamento aberto, insuficiência cardíaca

## Abstract

Relatamos o caso de um homem de 38 anos com fístula arteriovenosa traumática entre a artéria e a veia femorais comuns esquerdas, após ferimento por projétil de arma de fogo há 20 anos. Apresentava sintomas como úlcera de estase e alterações cardíacas há 4 anos. Na tomografia, identificou-se fístula e dilatação a jusante dos vasos ilíacos e da veia cava inferior, esta última medindo 37,5 mm. Como o paciente apresentava anatomia muito desfavorável e boa condição clínica, optou-se pela cirurgia aberta, com ótimos resultados. Devido ao risco de trombose venosa profunda e tromboembolismo pulmonar, optou-se pela implantação de filtro de veia cava e por anticoagulação perene. A cirurgia foi o tratamento definitivo para a insuficiência cardíaca e cicatrização da úlcera, restaurando a qualidade de vida do paciente. A via aberta foi uma ótima opção terapêutica, considerando a juventude do paciente, a distorção anatômica e a falta de material endovascular apropriado.

## INTRODUÇÃO

A fístula arteriovenosa (FAV) é uma comunicação anormal e permanente entre uma artéria e uma veia^1^ . O trauma penetrante é a causa mais comum de FAV traumática, seguido por lesões iatrogênicas e trauma contuso. Podem ocorrer alterações fisiopatológicas e estruturais, com desvio do fluxo sanguíneo da artéria para a veia, resultando em achados físicos característicos e repercussões clínicas para o paciente. Ao exame físico, podem ser observados frêmito e sopro, devido ao turbilhonamento do sangue, enquanto os pulsos periféricos podem estar fracos ou ausentes. O membro acometido pode apresentar palidez, cianose, edema, dermatite e, em casos mais avançados e graves, claudicação intermitente, ulcerações por estase venosa e gangrena. Alterações sistêmicas, como distúrbios do ritmo ou da função cardíaca, também podem estar presentes^2^ .

Apenas cerca de 2% das FAVs traumáticas apresentam resolução espontânea^3^ , o que significa que a correção cirúrgica é necessária em praticamente todos os casos. O tratamento endovascular é a melhor opção; contudo, em casos de anatomia desfavorável, a via aberta deve ser considerada^4^ .

Relatamos o caso de um paciente com FAV traumática no membro inferior esquerdo há 20 anos, que apresentava sintomas há 4 anos e foi tratado por meio de cirurgia aberta. O estudo foi aprovado pelo Comitê Nacional de Ética em Pesquisa sob número do parecer consubstanciado 6.578.832 e número do CAAE 74856423.3.0000.5404.

## RELATO DE CASO

### PARTE I: APRESENTAÇÃO DO CASO 

Homem, 38 anos, com queixa de dor, edema, dermatite e úlcera por estase venosa em face lateral do membro inferior esquerdo há 4 anos. Devido aos sintomas, não conseguia exercer suas atividades diárias nem trabalhar. Trata-se de paciente previamente hígido, trabalhador da construção civil, que sofreu trauma por projétil de arma de fogo, com orifício de entrada na região inguinal esquerda e saída na nádega ipsilateral, há 20 anos. À época, foi atendido em outro hospital, onde o projétil foi retirado e o paciente liberado, sem realização de exames de imagem ou seguimento ambulatorial.

Ao exame físico, apresentava massa pulsátil na região inguinal esquerda, associada a frêmito e sopro; edema em todo o membro, desde a raiz da coxa, que interferia demasiadamente na deambulação; e varizes e úlcera de estase na face lateral da perna esquerda. Além disso, havia presença apenas de pulso femoral, sem sinais isquêmicos (Figura 1). O paciente negava sintomas sugestivos de insuficiência cardíaca, como ortopneia ou dispneia paroxística noturna. Todavia, apresentava hepatomegalia dolorosa, o que sugeria insuficiência cardíaca.

**Figura 1:** A imagem digital mostra edema, dermatite ocre e úlcera de estase venosa em face lateral de perna esquerda do paciente.

Realizou-se angiotomografia computadorizada (TC), que evidenciou FAV entre a artéria femoral comum esquerda (AFCE) e a veia femoral comum esquerda (VFCE), com dilatação a jusante dos vasos ilíacos e da veia cava inferior (VCI), a qual apresentava cerca de 37,5 mm na junção das veias ilíacas (Figuras 2 e 3). Durante a internação, evidenciou-se *flutter* atrial sem instabilidade hemodinâmica no eletrocardiograma e insuficiência cardíaca de alto débito no ecocardiograma. Houve descompensação da insuficiência cardíaca, sendo necessária compensação cardiológica com medicações e reversão do *flutter* para ritmo sinusal por meio de cardioversão elétrica.

**Figura 2:** A reconstrução tridimensional da angiotomografia computadorizada mostra a dilatação dos vasos ilíacos, veia cava inferior, veias pélvicas e da região inguinal esquerda.

**Figura 3:** A angiotomografia computadorizada mostra a dilatação da veia cava inferior, com até 37,5 mm de diâmetro na junção das veias ilíacas.

### PARTE II: O QUE FOI FEITO? 

Em seguida, procedeu-se ao tratamento da FAV. Optou-se pela correção aberta, devido às dimensões dos vasos, à indisponibilidade de materiais endovasculares com dimensões suficientes e ao fato de o paciente ser jovem, hígido e apresentar boas condições clínicas. Acessou-se a região inguinal esquerda por inguinotomia transversa, com isolamento da AFCE e, parcialmente, da VFCE, dada a dificuldade de dissecção e presença de aderências. Devido ao calibre dos vasos, a proposta inicial foi inserir e insuflar um balão de acomodação na AFCE, acima da FAV, a fim de conter o fluxo para a veia e permitir a rafia da FAV. Entretanto, o balão insuflado não foi capaz de ocluir totalmente a artéria. Durante a dissecção, ocorreu lesão da VFCE em local de difícil clampeamento com instrumentais, sendo o sangramento controlado por pinçamento digital com o indicador e polegar.

Optou-se, então, por abertura do canal inguinal e do abdômen à esquerda, com acesso retroperitoneal, isolamento e clampeamento cirúrgico da artéria ilíaca externa esquerda (AIEE) e da veia ilíaca externa esquerda, obtendo-se controle do sangramento. Em seguida, realizou-se o reparo da FAV com sutura contínua de polipropileno 5-0, com cessação imediata do frêmito. Pelo tempo de evolução e pela fibrose, as paredes da artéria e da veia estavam aderidas, impossibilitando a dissecção, sendo necessário realizar o reparo da fístula englobando as duas paredes. Realizou-se a rafia da parede anterior da veia, lesionada durante a dissecção, também com sutura contínua de polipropileno 5-0. Realizou-se enxerto terminolateral entre a AIEE e a AFCE, utilizando veia safena magna invertida ipsilateral, devido ao comprometimento da parede arterial decorrente da FAV prolongada e à necessidade de sutura conjunta das paredes arterial e venosa para o fechamento da fístula (Figura 4).

**Figura 4:** A imagem digital mostra o intraoperatório com enxerto entre AIEE e AFCE com veia safena magna invertida ipsilateral. Nota-se dilatação de veia femoral comum esquerda. AFCE = artéria femoral comum esquerda; AIEE = artéria ilíaca externa esquerda; VFCE = veia femoral comum esquerda; VSMI = veia safena magna invertida.

Houve uma perda estimada de 4.000 mL de sangue durante a cirurgia, sendo o evento mais crítico a lesão da VFCE. Todavia, o paciente permaneceu hemodinamicamente estável, necessitando transfusão de hemoderivados, administração de solução cristaloide e uso de noradrenalina em baixas doses no intraoperatório.

A implantação de um filtro de VCI estava programada para o mesmo tempo cirúrgico; entretanto, isso não foi possível devido ao diâmetro da VCI. Iniciou-se anticoagulação plena com enoxaparina subcutânea, na dose de 1 mg/kg a cada 12 horas, com reavaliação por TC programada nas semanas seguintes à cirurgia, com o objetivo de avaliar o diâmetro da VCI.

O paciente foi extubado no primeiro dia de pós-operatório, a droga vasoativa foi retirada no segundo dia e o paciente recebeu alta da UTI no quarto dia. Na enfermaria, manteve-se anticoagulação plena, uso de meia elástica, deambulação estimulada, membro elevado ao ficar deitado e curativo na úlcera. Houve melhora significativa dos sintomas, da deambulação e da cicatrização da úlcera. A TC foi repetida na terceira semana de pós-operatório, mostrando redução significativa das dilatações das veias ilíacas e cava, com a última agora apresentando diâmetro de 24,7 mm no maior eixo (Figura 5). Essa evolução possibilitou o implante infrarrenal de filtro de VCI não removível Denali™ (33 mm) por acesso via veia femoral comum direita, sob anestesia local e sem intercorrências. O paciente recebeu alta no 23º dia de pós-operatório, com prescrição de rivaroxabana via oral 20 mg/dia, uso de meia elástica e encaminhamento para seguimento ambulatorial.

**Figura 5:** A angiotomografia computadorizada em corte transversal mostra a veia cava inferior de 24,7 mm de diâmetro em seu maior eixo, 3 semanas após a cirurgia.

Nas consultas realizadas no 1º e 3º meses de pós-operatório, o paciente estava sem dor, deambulando normalmente, sem hepatopatia dolorosa e com ritmo cardíaco em sinusal. A úlcera encontrava-se completamente cicatrizada no 3º mês (Figura 6). No 6º mês, o paciente estava assintomático, havia retornado às suas atividades laborais normalmente e o ecocardiograma estava normal. A ultrassonografia evidenciou oclusão do enxerto e dilatação venosa, porém com boa perfusão distal. A radiografia simples demonstrou o filtro de VCI bem alocado. Foi mantida anticoagulação com rivaroxabana e acompanhamento ambulatorial contínuo.

**Figura 6:** A imagem digital mostra a evolução da úlcera de estase venosa na face lateral de perna esquerda do paciente. Da esquerda para direita: antes da cirurgia, 3 semanas e 3 meses após a cirurgia.

## DISCUSSÃO

Na literatura, lesões penetrantes são as principais causas de FAVs traumáticas. Segundo Robbs et al., no manejo de 202 pacientes com FAVs traumáticas, as causas penetrantes representaram 98% dos casos, com os ferimentos por arma de fogo correspondendo a 26% deles. Desses pacientes, apenas três apresentaram insuficiência cardíaca, e todos tinham cardiomegalia subjacente^5^ . Esse dado reforça a raridade do comprometimento cardíaco. O paciente deste relato era previamente hígido e não possuía alterações cardíacas prévias. Provavelmente, o tempo prolongado da FAV culminou em alterações hemodinâmicas que resultaram em repercussões cardíacas, exigindo intervenção medicamentosa e cardioversão elétrica. O tratamento definitivo para essas alterações é cirúrgico. A literatura comprova que há reversão do quadro cardíaco após a cirurgia, sendo a ligadura o único tratamento definitivo capaz de alterar a sobrevida da paciente^3^ .

O aumento do fluxo sanguíneo redirecionado para a veia causa aumento da pressão intravascular, dilatação venosa, insuficiência valvar, estase sanguínea, alterações pigmentares e, em casos avançados, úlceras. Após a cirurgia, observou-se cicatrização da úlcera, melhora do edema, da dor, da deambulação e da qualidade de vida. Em consonância com a literatura, a cicatrização de úlceras por insuficiência venosa causado por FAV é facilmente alcançada após a cirurgia^2^ .

O tratamento endovascular é, em geral, a melhor opção para lesões vasculares, sobretudo em procedimentos complexos e potencialmente perigosos. O reparo aberto, por sua vez, possui morbimortalidade considerável, uma vez que há pressão venosa elevada e inflamação dos tecidos vizinhos, o que torna as condições locais hostis. Grandes vasos colaterais, com anatomia venosa complexa, são frequentemente envolvidos, podendo culminar em sangramentos vultuosos^4^ e até mesmo morte.

A anatomia do nosso paciente era muito desfavorável ao tratamento endovascular, com vasos extremamente dilatados e tortuosos. Consultou-se diversos fornecedores de próteses, mas nenhum possuía material com tamanho suficiente para os vasos do paciente. Por isso, e pelo fato de o paciente ser jovem e hígido, optou-se pela via aberta. O procedimento foi desafiador devido à intensa aderência local, sobretudo da VFCE, e à grande dilatação e tortuosidade dos vasos. A lesão da VFCE foi a principal responsável pelo sangramento intraoperatório, com necessidade de expansão volêmica com cristaloide, transfusão de hemoderivados e uso de noradrenalina. Considerando o estado clínico do paciente, houve boa resposta intraoperatória às medidas, com ótima recuperação pós-operatória.

Devido ao risco de trombose venosa profunda e tromboembolismo pulmonar, que ocorrem principalmente nos primeiros 30 dias de pós-operatório^6^ , optou-se por anticoagulação plena com enoxaparina subcutânea e implantação de filtro de VCI após a redução do diâmetro da VCI, ainda durante a internação. Na alta, foi prescrita rivaroxabana 20 mg/dia. Essa medicação é um anticoagulante oral que atua na inibição direta do fator Xa. Estudos como o *Einstein Extension* e *Einstein Choice* mostraram que seu uso prolongado, por até 12 meses, possui baixo risco de sangramento grave^7,8^ . Como o paciente apresentava baixo risco de sangramento, dilatação excessiva das veias e filtro de VCI não removível, optou-se inicialmente pelo uso da rivaroxabana na dose de anticoagulação. Durante o seguimento do paciente, será avaliada a possibilidade de redução do diâmetro das veias e da dose da rivaroxabana para 10 mg/dia (dose profilática), entre 6 e 12 meses após o procedimento.

De acordo com as Diretrizes Brasileiras de Diagnóstico e Tratamento de Lesões Vasculares Traumáticas, os traumas vasculares de membros em pacientes hemodinamicamente estáveis, com índice tornozelo-braquial (ITB) menor que 0,9, possuem indicação de exame de imagem, sendo que a angiotomografia é o padrão-ouro. Em traumas penetrantes com ITB maior ou igual a 0,9 e na ausência de sinais de lesão vascular, pode-se dar alta sem exame de imagem, desde que haja acompanhamento ambulatorial, devido à possibilidade de manifestações tardias^9^ .

Uma das lições deste caso é que o cirurgião não deve perder o diagnóstico de FAV nos casos de trauma penetrante em trajetos de vasos, mesmo que haja presença de pulso e ausência de sangramento, a fim de evitar complicações tardias. A realização de angiotomografia e o seguimento ambulatorial são imprescindíveis para o diagnóstico precoce e tardio, respectivamente.

Conclui-se que a ligadura da FAV traumática foi o tratamento definitivo para as repercussões clínicas, como úlcera e insuficiência cardíaca, restaurando a qualidade de vida do paciente. A via aberta constituiu ótima opção terapêutica, devido ao fato de o paciente ser jovem, apresentar distorção anatômica e à falta de material endovascular apropriado.
